# First Immunodetection of Sensory and Nervous Systems of Parasitic Larvae (Glochidia) of Freshwater Bivalve *Nodularia douglasiae*

**DOI:** 10.3389/fphys.2022.879540

**Published:** 2022-04-11

**Authors:** Viktoria E. Nikishchenko, Elena M. Sayenko, Vyacheslav A. Dyachuk

**Affiliations:** ^1^ A. V. Zhirmunsky National Scientific Center of Marine Biology, Far Eastern Branch, Russian Academy of Sciences, Vladivostok, Russia; ^2^ Federal Scientific Center of the East Asia Terrestrial Biodiversity, Far Eastern Branch, Russian Academy of Sciences, Vladivostok, Russia

**Keywords:** freshwater mollusk, glochidia, parasitic larvae, hair cell, neurotransimitter, serotonin, FMRF—amide, adductor muscle

## Abstract

Most freshwater mussels have an unusual life cycle that requires host fish species for larval (glochidia) development and dispersal. Glochidia have a unique morphological structure that adapts to parasitic lifestyles and survival. The morphology of the glochidial shells of most Unionoidea, a group of freshwater bivalve mollusks, has been studied in detail using light and scanning electron microscopy. This study summarizes our data on the glochidia shell morphology of the Asian mussel *Nodularia douglasiae* from two localities in the Primorsky Territory, the Russian Far East. In contrast to the shell morphology of glochidia, little is known about the neurodevelopment of the Unionoidea. Herein, we first demonstrate that the structures of the sensory, muscle, and nervous systems of the glochidia larvae of *N. douglasiae* differ dramatically from those of the comparable larval systems of marine bivalve species, as revealed through alpha-acetylated tubulin, serotonin (5-HT), and FMRFamide antibodies as well as phalloidin for detection of F-actin and whole-mount confocal microscopy. We found that the glochidia sensory system included four pairs of tubulin-lir multicilia hair cells. Non-ciliar tubulin-lir cells synthesize the neuropeptide FMRFamide and are identified as afferent neurons collecting information from peripheral tubulin-lir hair sensory cells to nervous regulators. The glochidia’s muscular system was represented by a smooth adductor, retractors, and minor muscle bundles associated with the shell and visceral organs. The 5-HT-lir larval system is arranged most simply and consists of two immunopositive neurons innervating the adductor. The FMRFamide-lir system is more complicated and consists of several neuronal centers comprising neuronal bodies and their neurites in different areas of the larva. The FMRFamide-lir neurons are closely associated with sensory hair cells, and others, together with 5-HT-lir neurons, may be involved in the anlagen of adult ganglia. Thus, the nervous system of *N. douglasiae* glochidia is drastically different from other mollusks and lophotrochozoans because of the absence of an apical organ and the location and composition of FMRFamide and 5-HT cells. Morphological, molecular, and behavioral investigations of Unionoidea taxa need to be further conducted to investigate the parasite-host relationship, nerve-dependent regulation of parasite behavior, and evolution of mollusks.

## Introduction

Freshwater species of bivalve mollusks have evolved various life cycle traits adapted to parasitizing obligate hosts. The parasitic larval stage of some freshwater mussels, the glochidia, can temporarily attach to the external surface of a suitable host (fish and some amphibians); thus, nutrition and dispersal of the parasite larvae are provided (Lefevre and Curtis, 1910; Young, 1911; Arey, 1921, 1932; Kat, 1984; Watters and O’Dee, 1998; Wächtler et al., 2001; Barnhart et al., 2008; Walker et al., 2014; Miyahira et al., 2017).

Although the shape and morphology of glochidia may vary among taxa, the general structural plan of unionid glochidia is similar. In addition to the adult bivalve, the glochidium has two valves and a dorsal hinge. The ventral tip of each valve can often be armed with a hook that varies in size and shape (Hoggarth, 1999; Wächtler et al., 2001; Sayenko, 2006). Hookless larvae usually attach to fish gills of a reduced number of hosts (Araujo et al., 2002; Barnhart et al., 2008).

The Unionoidea is an ecologically diverse and geographically widespread group of freshwater bivalve mollusks, including six families (Unionidae, Margaritiferidae, Hyriidae, Etheriidae, Mycetopodidae, and Iridinidae) (Graf, 2013). The morphology of unionid shells is the subject of active taxonomic studies (Klishko et al., 2017; Lopes-Lima et al., 2020; Sayenko et al., 2021). The glochidial shell consists of two layers. The inner layer is thick and punctuated by pores, although their outer ends are covered by a thin outer layer (Kinzelbach and Nagel, 1986) that forms a special external microstructure. Under high magnification, ridges and depressions, which appear to be organized regularly, can be observed on the external shell surface (Hoggarth, 1999; Sayenko, 2016; Sayenko et al., 2021).

Once attached to the mucous membrane of fish gills or other structures, the glochidia are encapsulated into a parasitic cyst owing to epithelial hyperplasia and lamellar fusions (Treasurer and Turnbull, 2000; Fisher et al., 2002; Howerth and Keller, 2006; Rogers-Lowery and Dimock, 2006; Ieshko et al., 2016).

In intensive parasitosis of the gills, there may be general deterioration in fish condition (Nezlin et al., 1994; Thomas et al., 2013), respiratory distress (Thomas et al., 2013), growth reduction (Ooue et al., 2017), and behavioral changes (Horký et al., 2019). However, after the glochidia separates from the host fish, the gills can restore their integrity without affecting the fish condition (Treasurer and Turnbull, 2000). There is also a complete skin restoration after infection of fish skin with glochidia (Rogers-Lowery and Dimock, 2006).

A few species of Unionidae are occasionally or permanently simultaneous hermaphrodites; however, in most cases, unionid sexes are separate (Bauer, 1987). Unionid embryonic development occurs in the outer (marsupial) gill demibranchs of female mussels (Graf and Ó Foighil, 2000; Wächtler et al., 2001). As a result of embryo cleavage, a coeloblastula is formed (Ivanova-Kazas, 1977). Then, a small gastric invagination turns into a small endodermal sac after the blastopore closure. Finally, the shell gland is determined in the usual way for bivalves, and the shell has an unusually thick cap-like shape with numerous pores (Wood, 1974).

The inner surface of the glochidial valves is covered with larval mantle-bearing bundles of sensory hairs along the mantle edge. Unlike adult unionids, the glochidium has only one adductor muscle that degenerates during metamorphosis and is replaced by a pair of adductors in a juvenile mussel. The following organs are rudimentary in the glochidium: the gills, intestines, and foot. Internal organs such as the mouth, anus, and digestive tract are absent in the glochidium and appear in the juvenile due to metamorphosis (Wood, 1974; Ivanova-Kazas, 1977). To successfully attach to the host fish, the glochidia of some taxa must have a long sticky larval thread. There are some variations in the form, position, and presence or absence of the larval thread, even in closely related species (Wächtler et al., 2001).

*Nodularia douglasiae* (Griffith and Pidgeon, 1833) is a widespread freshwater mussel from the family Unionidae, and its distribution extends from Vietnam in the south to the Russian Far East in the north. It also includes south of the Korean Peninsula, Kyushu, and Honshu islands in Japan (Graf and Cummings, 2007; Klishko et al., 2017, 2018; Lopes-Lima et al., 2020). Such a wide distribution and relatively high abundance of *N. douglasiae* make it an evaluable species for studies of the phylogeny and evolution of mussel taxa (Klishko et al., 2017, 2018; Sano et al., 2017; Choi et al., 2020; Lopes-Lima et al., 2020; Sayenko et al., 2021), comparative internal (organs/tissues) and external (shell, teeth, hinge) gross anatomy (Sano et al., 2017; Sayenko et al., 2021), histology (Sayenko and Rasshchepkina, 2018), the behavior of both larvae and adult mussels (Liu et al., 2006; Taeubert et al., 2012; Itoh et al., 2021), and morphology of glochidial shells (Inaba, 1941, 1964; Park and Kwon, 1993; Wu et al., 1999; Sayenko, 2006, 2008, 2015) among other aspects. First, brief descriptions of *N. douglasiae* glochidia were made using light microscopy for mussels from Korea (Inaba, 1941) and the Russian Far East (Antonova and Starobogatov, 1988). Later, the glochidial shell morphology was investigated using light and scanning electron microscopes (Park and Kwon, 1993; Wu et al., 1999; Sayenko, 2015).

To understand the physiology and parasitic behavior of the glochidium, it is necessary to investigate certain aspects of the anatomy and morphology of the nervous systems in glochidia, the presence which in larvae has caused many doubts. The lack of information concerning the metamorphosis of the nervous system in freshwater mussels led us to examine this question more closely. In this study, we examined the localization of transmitters in the nervous system and sensory structure of *N. douglasiae* larvae for the first time using whole-mounting immunostaining techniques and confocal microscopy. In addition, the specialized neurostructures of this larva have been described and compared with those of other planktotrophic larvae (veligers) of Bivalvia.

## Materials and Methods

### Specimen Sampling

**FIGURE 1:**
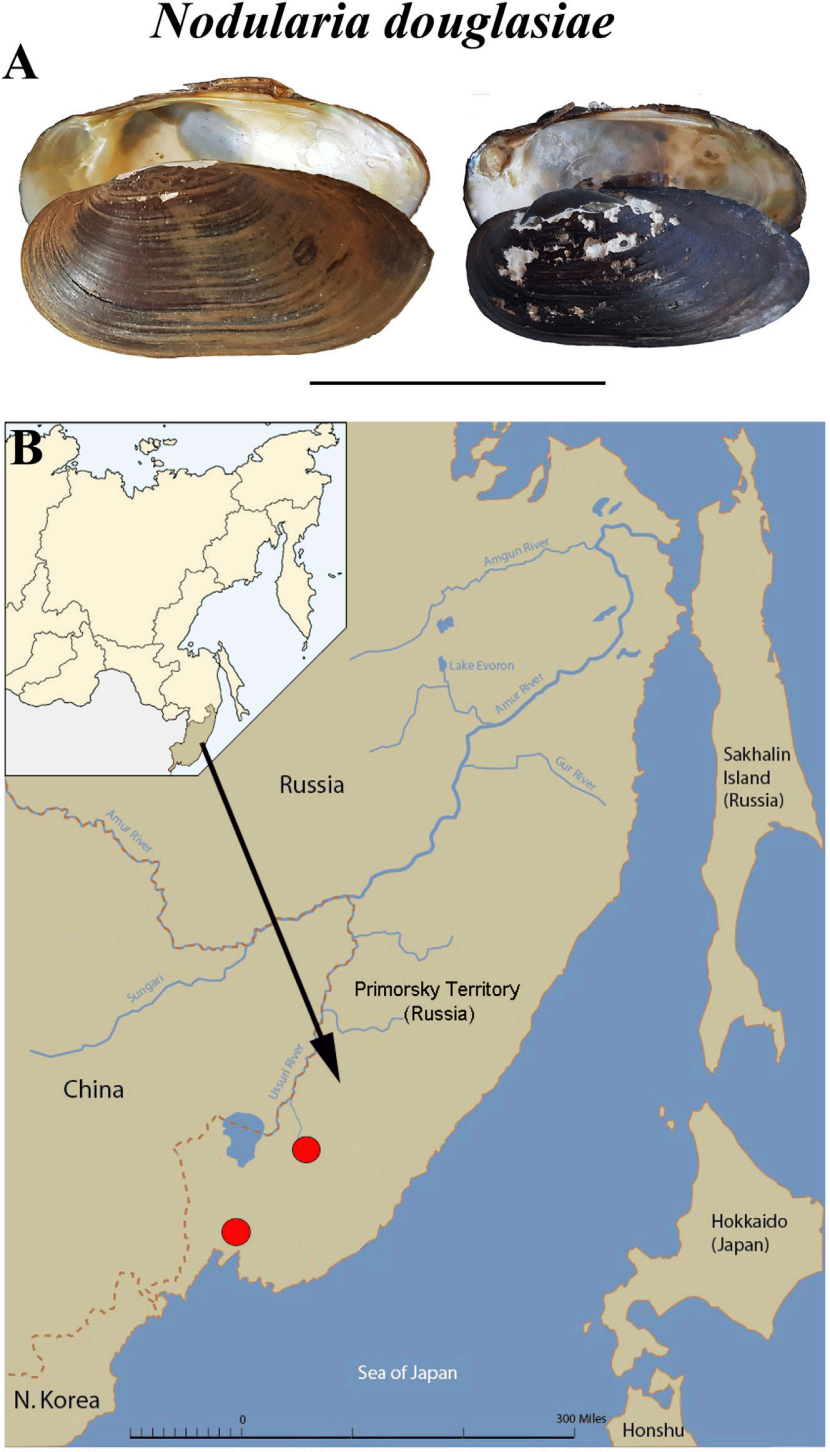
Mussel *Nodularia douglasiae:* mollusk shells and scheme of the life cycle. **(A)** Shells of adult freshwater bivalve *N. douglasiae*. Scale bar 5 cm. **(B)** Map of the freshwater mussel collection region. The arrow points to the material collection region (Primorsky Krai) in Russia.

Specimens of *Nodularia douglasiae* (Figure 1A) were collected from two localities in the Primorsky Territory, Russian Far East (Razdolnaya River, N43.555483, E131.905971, and Arsenievka River, N44.845826, E133.578503) (Figure 1B). Totally, 21 adult mussels with glochidia were collected and investigated. Samples were collected once a month from May to October, 2020. Adult mollusks were picked up by hand at a shallow depth (0.5–1.5 m). The presence of glochidia in the outer demibranchs of adult mussels was checked immediately in the field, the degree of glochidia maturity was finally determined in laboratory under stereomicroscope. The degree of glochidia maturity was indicated by the shape and colour of the outer demibranchs in adult mussels, so as by the flapping movements of live glochidia (Hoggarth, 1999; Sayenko, 2006; Sayenko and Kazarin, 2022).

**FIGURE 2:**
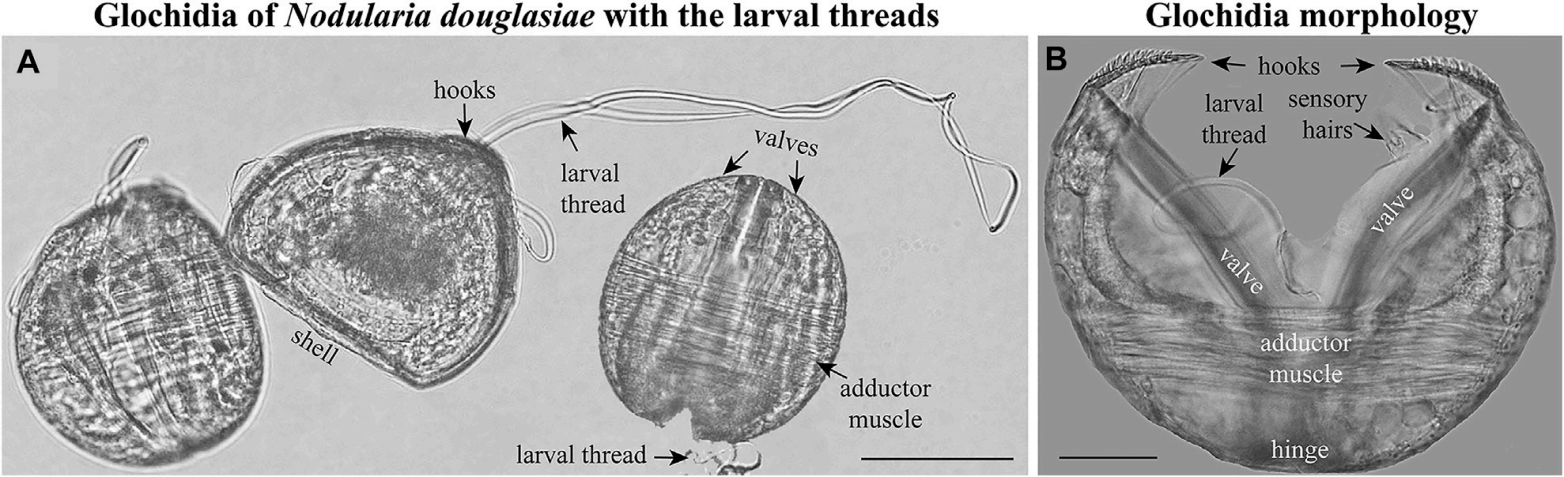
Glochidia of *Nodularia douglasiae.* **(A)** Mature glochidia of *N. douglasiae* with the larval threads. **(B)** Glochidium with open valves. Large styliform hooks, adductor muscle, and bundles of sensory hairs (under the hooks), partly the larval thread, are visible. Light microscopy. Collection: Amur River basin. Scale bars, **(A)**, 100 μm; **(B)**, 50 μm.

**FIGURE 3:**
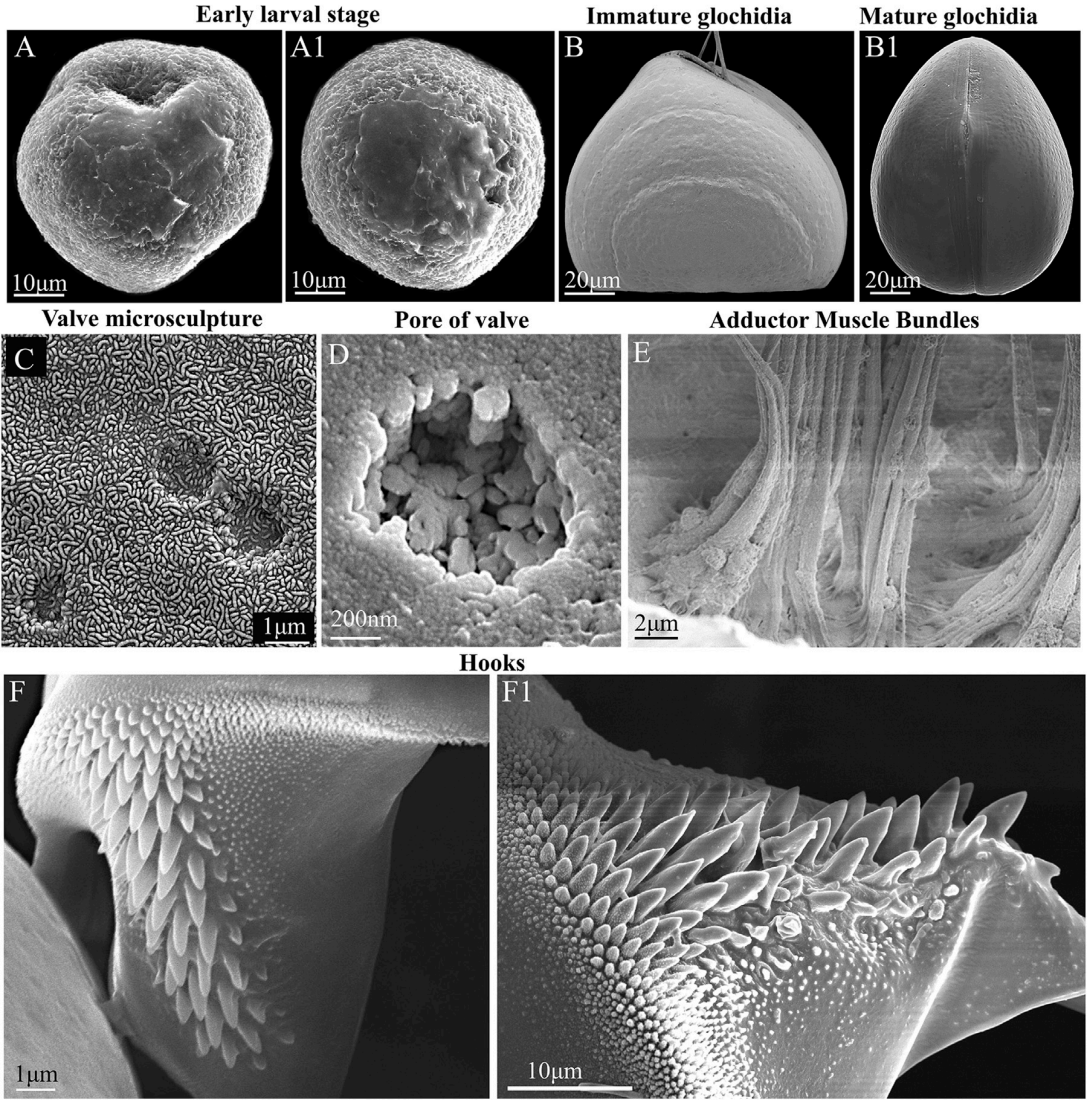
Glochidia of *Nodularia douglasiae:* stages of development, external and internal morphology. **(A,A1)** Early larval stage. **(B)** Immature glochidium. The growth lines of the shell are visible; the immature larva does not yet have hooks (without potassium hydroxide treatment) at this stage of development. **(B1)** Mature glochidium, closed shell. **(C)** Exterior valve microsculpture at the center of the valve. This is the central part of the valve; three non-through pores are visible. **(D)** Pore on the inner surface of the valve. **(E)** Muscle bundles of the smooth adductor. The photo was taken through the slightly open valves gap; the edge of the shell is visible from below. **(F,F1)** Hook. SEM. Collection: Amur River basin. Scale bars, **(A,A1,F1)**, 10 μm; **(B,B1)**, 20 μm; **(C,F)**, 1 μm; **(D)**, 200 nm; **(E)**, 2 μm.

**FIGURE 4:**
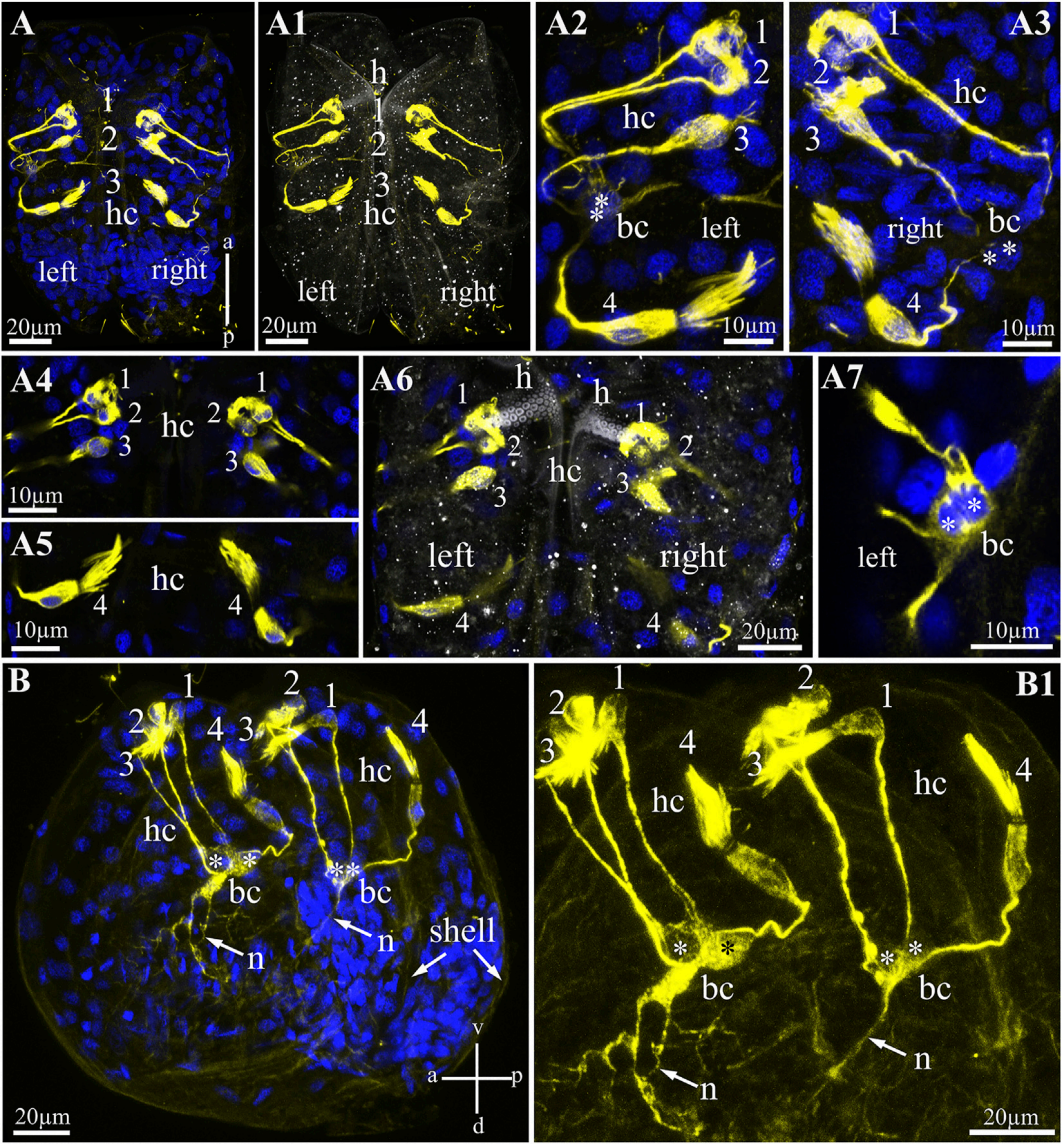
Hair cells in glochidia of *Nodularia douglasiae*. **(A)** Whole-mount larva immunostained with anti-acetylated tubulin (yellow) antibody and DAPI (blue). Posterior view. **(A1)** The same larva in transmitted light and tubulin. **(A2,A3)** Higher magnification of tubulin-lir hair cells (left, A2 and right, A3). **(A4,A5)** Inset of hair cells separately a bundle of three cells **(A4)** and a separately lying hair cell **(A5)**; **(A6)** separate Z-stack showing the position of the hair cells relative to the larval hook; **(A7)** tubulin-lir basal cells; vertical a-p labelled line refers to anterior-posterior body axes. **(B,B1)** whole-mount larva, lateral view; **(B)** larva stained by tubulin with DAPI; **(B1)** larva stained only by tubulin. Vertical v-d labelled line refers to ventro-dorsal body axes and horizontal line a-p labelled line refers to anterior-posterior body axes of larva. Abbreviations: bc, basal cells; h, hook; hc, hair cells; n, neurites. Scale bars, **(A,A1,A6,B,B1)**, 20 μm; **(A2–A5,A7)**, 10 μm.

**FIGURE 5:**
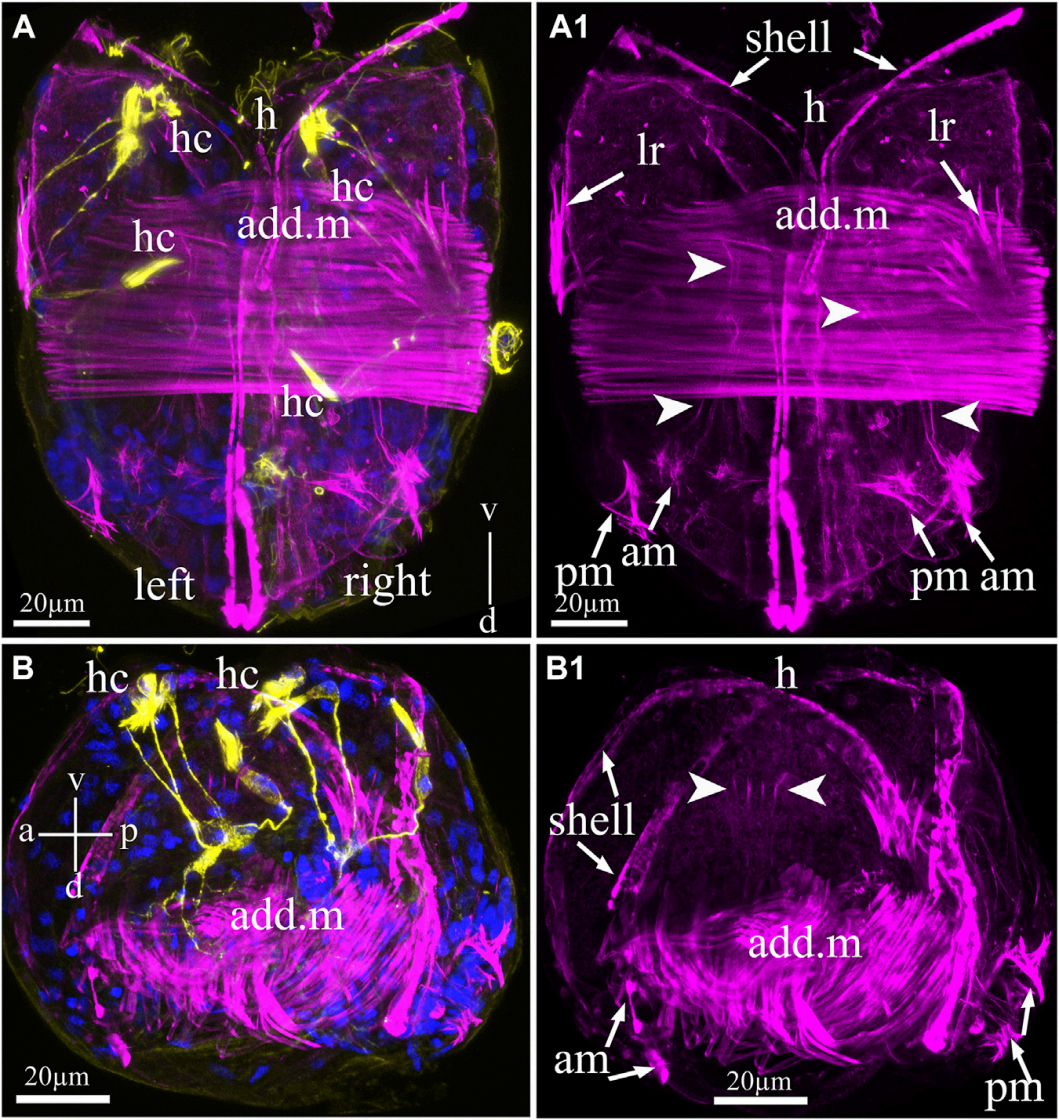
Myoanatomy of *Nodularia douglasiae* glochidia. **(A,A1)** Posterior view. **(B,B1)** Lateral view. The specimen was observed using confocal images produced by phalloidin staining (magenta) to visualize the larval muscle systems **(A–B1)** and anti-acetylated tubulin (yellow) antibody **(A,B)** together with DAPI **(A,B)**. **(A,A1,B,B1)** Confocal micrograph of the larval musculature. Arrowheads point to smooth massive muscle (adductor, add. m) in the central part of the larva, while arrows indicate that larval retractors (lr) are located on the edges of the shell (left and right) and anterior and posterior muscle bundles (AM and PM) **(A1,B1)**. **(B1)** On the lateral side of the larva, it is possible to distinguish separate muscle fibers indicated by arrowheads. **(A)** Vertical v-d labelled line refers to ventro-dorsal body axes. **(B)** Vertical v-d labelled line refers to ventro-dorsal body axes and horizontal line a-p labelled line refers to anterior-posterior body axes of larva. Abbreviations: add. m, adductor muscle; am, anterior muscle; h, hook; hc, hair cells; lr, larval retractors; pm, posterior muscle. Scale bars, **(A–B1)**, 20 μm.

**FIGURE 6:**
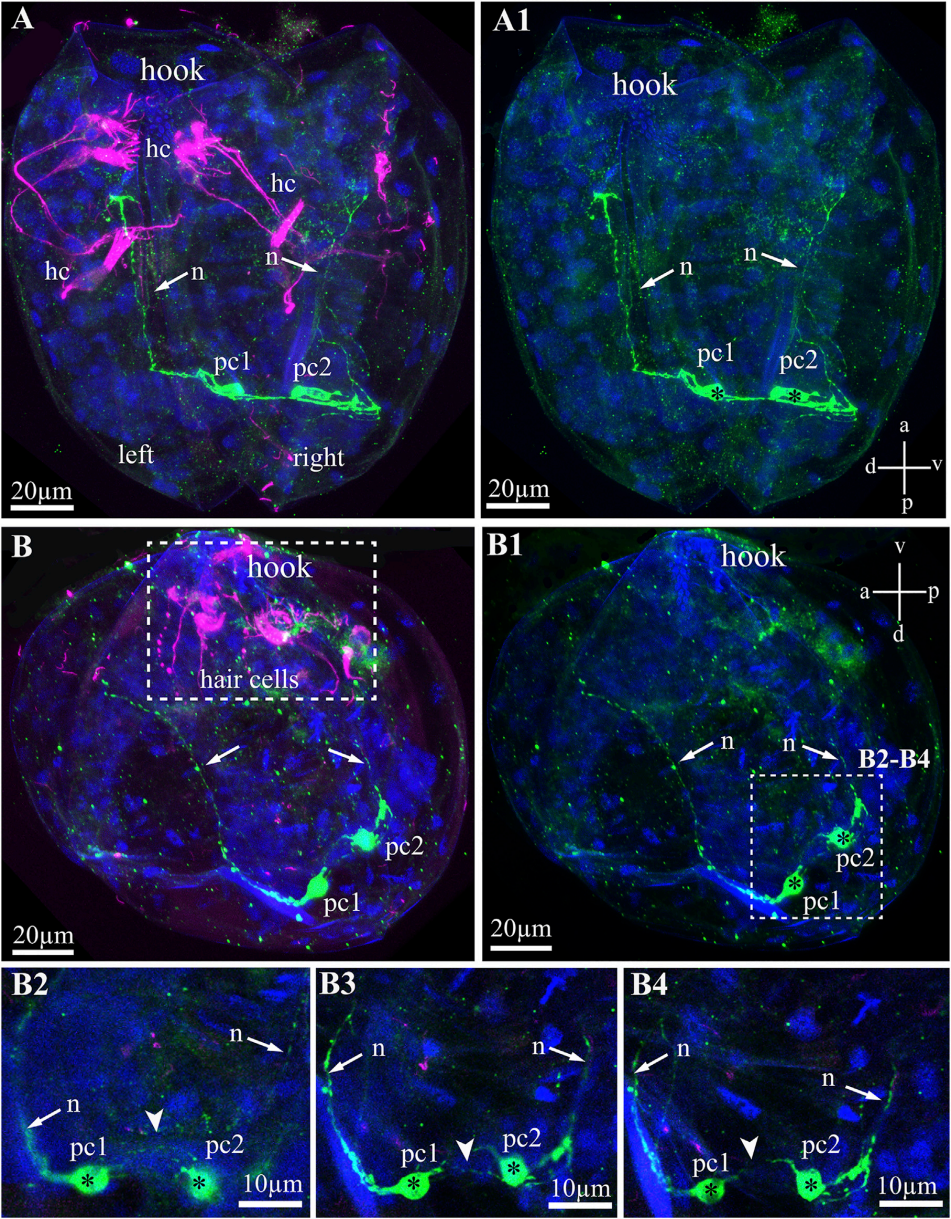
Serotonin-like immunoreactivity (5-HT-lir) in the *Nodularia douglasiae* glochidia. **(A,A1)** posterior view; **(B,B1)** lateral view. Green—5-HT-lir **(A–B1, B2–B4)**; magenta—hair cells, acetylated tubulin immunoreactivity **(A,B,B2–B4)**. **(A,A1,B,B1)** two 5-HT-lir cells on the posterior part of larva; **(B2,B4)** insets**:** high magnification of the two 5-HT-lir cells and their neurites running to the anterior part of larva. **(A1)** vertical a-p labelled line refers to anterior-posterior body axes and horizontal line d-v labelled line refers to dorso-ventral body axes. **(B1)** vertical v-d labelled line refers to ventro-dorsal body axes and horizontal line a-p labelled line refers to anterior-posterior body axes of larva. Abbreviations: hc, hair cells; n, neurites, pc, posterior cells. Scale bars, **(A–B1)**, 20 μm; **(B2–B4)**, 10 μm.

**FIGURE 7:**
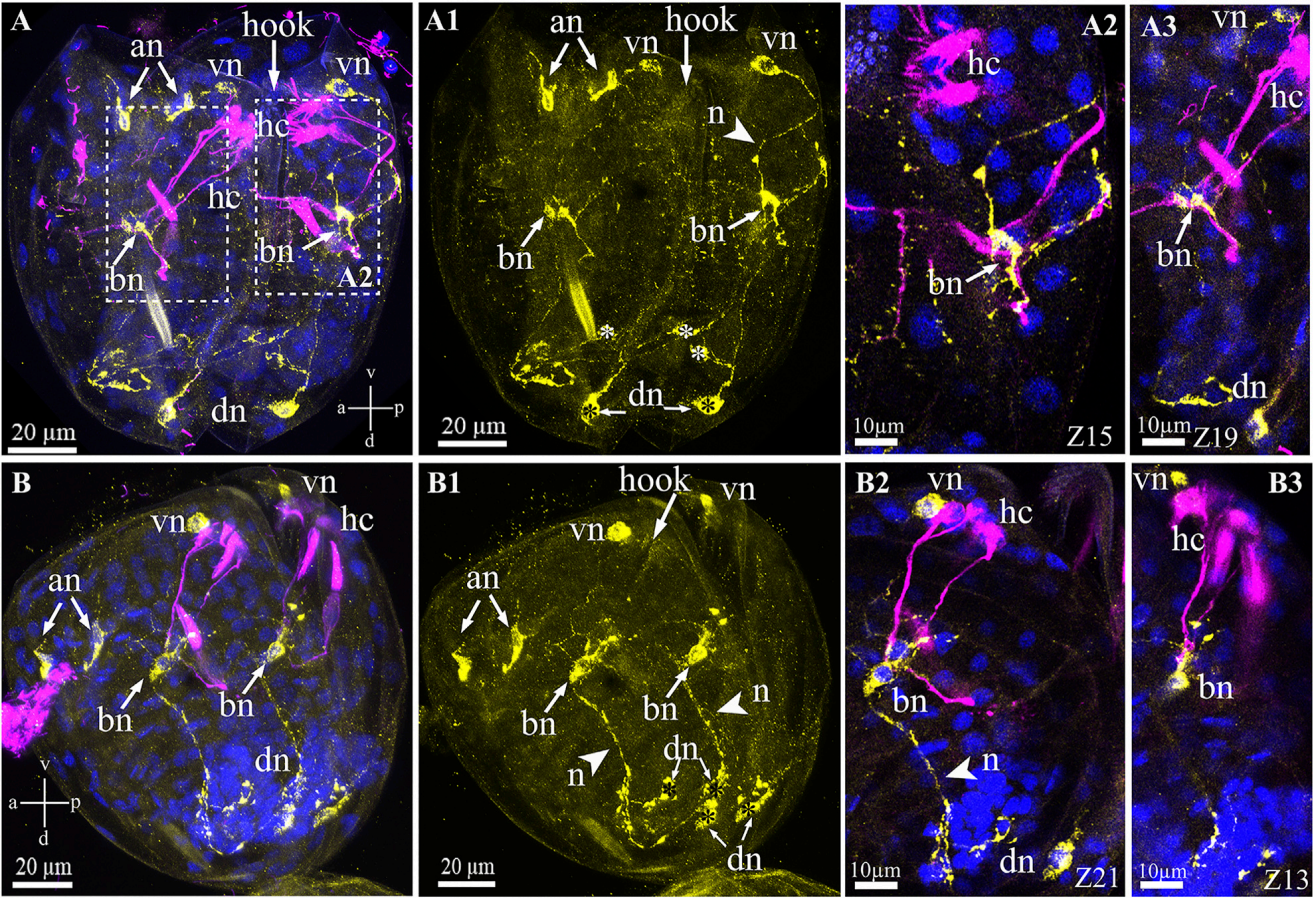
FMRFamide-like immunoreactivity (FMRFa-lir) in the *Nodularia douglasiae* glochidia. **(A,A3)** posterior view; **(B–B3)** lateral view. Yellow—FMRFa-lir; magenta—hair cells, acetylated tubulin immunoreactivity; blue—nuclei, DAPI. **(A,A1,B,B1)** FMRFamidergic nervous system larvae consist of paired anterior, ventral, dorsal, and basal neurons (arrows). The latter communicate both with ventral and dorsal neurons *via* neurites. Paired anterior neurons do not contact other neurons or hair cells of glochidia. **(A2,A3,B2,B3)** High magnification of contacts hair cells, basal cells, and ventral neurons. The insert shows that basal cells are double-positive for FMRFamide and acetylated tubulin (arrows). **(A,B)** Vertical v-d labelled line refers to ventro-dorsal body axes and horizontal line a-p labelled line refers to anterior-posterior body axes of larva. Abbreviations: an, anterior neurons; bn, basal neurons; dn, dorsal neurons; hc, hair cells; n, neurites; vn, ventral neurons; Scale bars, **(A,A1,B,B1)**, 20 μm; **(A2,A3,B2,B3)**, 10 μm.

**FIGURE 8:**
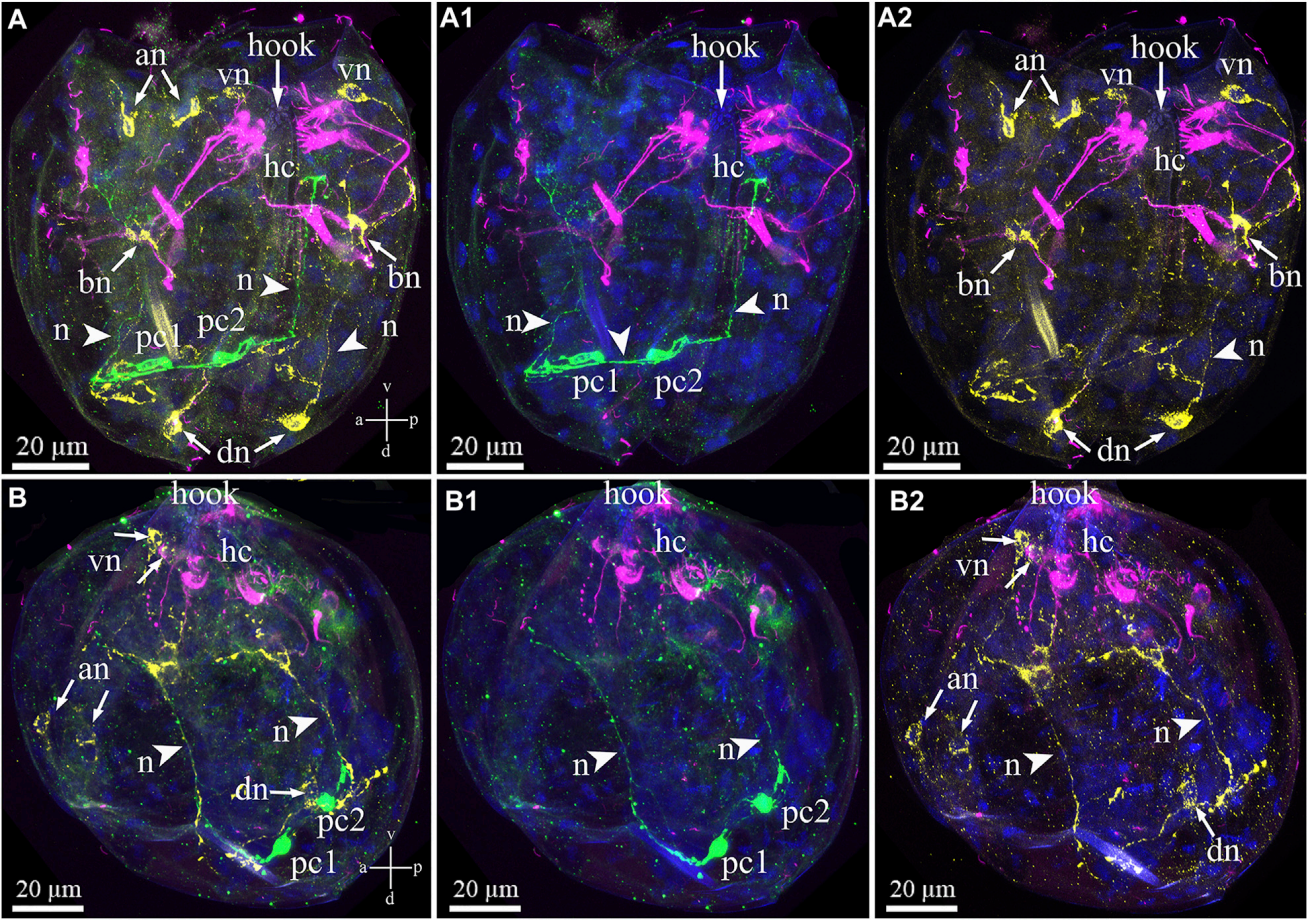
Double staining of 5-HT/FMRFa-lir nervous elements combined with tubulin immunoreactivity of hair cells in the *Nodularia douglasiae* glochidia. **(A–A2)** posterior view; **(B–B2)** lateral view. Yellow—FMRFa-lir; magenta—hair cells, green—serotonin, and blue—nuclei, DAPI. **(A, B)** Triple staining (FMRFa-lir/hair cells/5-HT); **(A1, B1)** double staining (hair cells/5-HT); **(A2,B2)** double staining (hair cells/FMRFa-lir); **(A,B)** 5-HT-lir posterior neurons and FMRFa-lir neurons are not colocalized as well as 5-HT-lir posterior neurons are not colocalized tubulin. Colocalization of immunostaining was detected only in combination FMRFa-lir/hair cells and only in base cells (neurons). The 5-HT-lir cells are connected to each other by neurites (arrowheads), and FMRFamidergic neurons connect with each other. **(A,B)** Vertical v-d labelled line refers to ventro-dorsal body axes and horizontal line a-p labelled line refers to anterior-posterior body axes of larva. Abbreviations: an, anterior neurons; bn, basal neurons; dn, dorsal neurons; hc, hair cells; n, neurites, pc, posterior cells; vn, ventral neurons . Scale bars, **(A–B2)**, 20 μm.

Glochidia of *N. douglasiae* were used to investigate the morphology of glochidial shells using light (Figure 2), scanning electron (Figure 3), and confocal microscopy (Figures 4–8). The collections of dry shells and samples of gills with glochidia preserved in 75% ethanol were stored at the Laboratory of Freshwater Hydrobiology, Federal Scientific Center of the East Asia Terrestrial Biodiversity, Far Eastern Branch of the Russian Academy of Sciences, Vladivostok (FSCEATB FEB RAS, hereafter). After removing the soft parts of the adult mussels, the shells were washed, dried, and labeled in the laboratory. Identification was performed using the most recent taxonomic revisions (Klishko et al., 2017; Lopes-Lima et al., 2020) and confirmed by DNA data of mussels collected from the two localities mentioned above (Sayenko et al., 2021).

### Procedures for the Light and Scanning Electron Microscopy

Samples with glochidia initially fixed in 75% alcohol were used to investigate the morphology of glochidial shells. Standard procedures were used to prepare larval shells for light and scanning electron microscopy (Hoggarth, 1999; Sayenko, 2006; Sayenko and Kazarin, 2022). To prevent any deformation or destruction, only chemical cleaning was used. First, the glochidia were washed several times in distilled water and cleaned of the soft tissues in a 5% KOH solution for 1.5–2 h; after that, the cleaned shells were washed several times in distilled water and dehydrated in an alcohol series (80%, 90%, and 96%), and then glochidial shells were mounted on permanent slides for light microscopy as well as on stubs for scanning electron microscopy and photography. For scanning electron microscopy, sputter coating with chromium or carbon was used. Photographs of glochidia were obtained using a Nikon light microscope (Nikon, Japan), Zeiss EVO 40, and Zeiss MERLIN scanning electron microscopes at the Biology and Genetic Engineering Center for Collective Use of FSCEATB FEB RAS.

The definition of the anterior and posterior regions of the glochidial valves follows that of Hoggarth (1987). Measurements were mainly made under light microscope and carried out according standard features (Hoggarth, 1999; Sayenko, 2006). Glochidial valve length was measured parallel to the hinge as the greatest distance from anterior to posterior margins. Glochidial valve height was measured perpendicular to length as the greatest distance from dorsal to ventral margins. In glochidium the hinge ligament extends the entire length of hinge, so hinge length (or hinge ligament length) was measured in a straight line from the anterior to posterior points of intersects. Hook length was measured from the tip to the distal end of the hook.

### Immunostaining Procedure

A 7.5% MgCl2 solution was added to *N. douglasiae* glochidia for muscle relaxation. Gills with larvae from adult mollusks were fixed in 4% PFA solution in phosphate buffer (PBS, 100 mM Na_3_PO_4_, 140 mM NaCl, pH 7.4) for 2–3 h at room temperature (Dyachuk and Odintsova, 2009). The fixed larvae were isolated from the gills and washed in 0.1 M phosphate-buffered saline (PBS). The samples were dehydrated using methanol solutions with increasing concentrations (25%, 50%, 75%, 100% methanol) and stored in 100% methanol at −20°C. Immediately before immunostaining, the larvae in 100% methanol were transferred to 0.1 M PBS by changing the solutions with a decreasing methanol concentration. The samples were incubated 1 h in 5% ethylenediaminetetraacetic acid (EDTA) in PBS at room temperature for shell decalcification, which is necessary to reduce the adhesion of antibodies to the shell and improves the quality of immunostaining (Voronezhskaya et al., 2008; Dyachuk and Odintsova, 2009; Yurchenko et al., 2019). The samples were rinsed in PBS supplemented with 0.1% TritonX-100 (PBST) for 3 × 30 min with agitation. Then, the samples were incubated at night in a blocking solution (10% donkey normal serum, 1% bovine serum albumin, and 1% Triton TritonX-100, 0.003% NaN_3_ in 0.1 M PBS) at +4°C. To detect the nerve structure, the larvae were incubated with primary antibodies (anti-serotonin and anti-FMRFamide rabbit polyclonal antibodies, ImmunoStar; serotonin goat polyclonal antibodies, ImmunoStar) a dilution of 1:1,000 together with monoclonal mouse antibodies raised against acetylated α-tubulin (ThermoFisher) in the blocking solution for 3 days at +4°C. Then, after washing in PBS (5 × 10 min), the samples were incubated overnight at 4°C in a secondary Alexa Fluor 488 donkey anti-goat IgG (DAG) (Invitrogen, A32814), Alexa Fluor 555 donkey anti-rabbit IgG (DAR) (Invitrogen A32794), Alexa Fluor 555 donkey anti-goat IgG (DAG) (Invitrogen, A32816), Alexa Fluor 488 donkey anti-rabbit IgG (DAR) (Invitrogen, A32814), Alexa Fluor 647 donkey anti-mouse IgG (DAM) (Invitrogen, A32787) with a dilution 1:1,000 and 0.1 μg/ml DAPI in the blocking solution. The larvae were then washed with PBST (5 × 20 min). All the specimens prepared for confocal microscopy were mounted on glass slides in a drop of 70% glycerol.

### Antibodies

In this study, we used rabbit or goat polyclonal antibodies against serotonin coupled to bovine serum albumin (BSA) with PFA (ImmunoStar Incorporated; Cat. No. 20080 and 20079). The manufacturer states that staining with these antisera is eliminated by pretreatment with 25 μg of the same serotonin–BSA conjugate per 1 ml of diluted antibody. For controls, we showed that pre-incubation of the antibody with the same conjugate (10 μg/ml, ImmunoStar, Cat. No. 20081) at 4°C overnight eliminated all immunolabeling of serotonin in the tissues. The preadsorption of the diluted antiserum with 10 mg/ml BSA overnight at 4°C did not influence this staining, i.e., these antibodies recognized only serotonin and not BSA. Data from other groups have shown that these antibodies detect serotonin in adult bivalves and their planktotrophic larvae (Voronezhskaya et al., 2008; Dyachuk and Odintsova, 2009; Dyachuk et al., 2015; Battonyai et al., 2018; Pavlicek et al., 2018; Yurchenko et al., 2019; Kotsyuba et al., 2020).

In addition to serotonin antibodies, we used an FMRFamide antibody to detect nervous system elements in the glochidia. FMRFamide-like peptides have been identified in the central and peripheral nervous systems of various taxonomic groups of mollusks (Price and Greenberg, 1977; Boer et al., 1980; Painter and Greenberg, 1982; Schot and Boer, 1982; Voronezhskaya et al., 2008; Dyachuk and Odintsova, 2009; Zatylny-Gaudin and Favrel, 2014; Battonyai et al., 2018; Yurchenko et al., 2019; Kotsyuba et al., 2020). The antiserum employed in this study was generated in rabbits against synthetic FMRFamide (Phe-Met-Arg-Phe-amide) conjugated to bovine thyroglobulin (Immunostar Incorporated; Cat. No. 20091). According to published data, this antibody reacts with FMRFamide in various animals, including mollusks, such as gastropods, bivalves, and polyplacophorans (Dyachuk et al., 2015; Wanninger, 2015; Yurchenko et al., 2019; Kotsyuba et al., 2020), indicating that the antigen has been evolutionarily conserved across a broad range of species. Specifically, the manufacturer confirmed that these antibodies react with antigens in caenogastropods, chitons, gastropods (*Helix pomatia*, *Aplysia* sp., *Ilyanassa obsoleta*, *Lymnaea stagnalis*, *Mopalia muscosa*, and abalones), and bivalves (*Mytilus trossulus*).

To detect the ciliar structure in glochidia, we used an acetyl-alpha tubulin (Lys40) monoclonal antibody (clone 6-11B-1 from Thermo Fisher, Cat. No. # 32-2700). Acetylation of α-tubulin is a modification in different microtubule structures and various eukaryotic cells. Currently, the published species include dogs, algae, rats, mollusks, zebrafish, humans, mice, and chickens, according to product-specific information from the company. Immunogen for preparation of primary antibody isolated from acetylated alpha-tubulin from the axoneme of sea urchin sperm flagella. Acetylation of alpha-tubulin is widely used to detect ciliary structures in annelid, mollusk, and nemertine larvae (Wanninger, 2015; Yurchenko et al., 2019; Magarlamov et al., 2020). In addition, these antibodies mark neuronal elements in some adult mollusks and annelids (Faller et al., 2012; Kotsyuba et al., 2020; Temereva et al., 2021). Therefore, mollusk larvae stained only with secondary antibodies were analyzed as controls for all primary antibodies.

### Confocal Microscopy

Samples of larvae stained by immunocytochemical staining were scanned using an LSM 780 confocal microscope (Zeiss, Germany) and Zen software using lasers with the following wavelengths: 405, 488, 555, and 647 nm. All images of the larvae were obtained in the Z-stack mode with an optical slice thickness of 1 μm along the *Z*-axis. The obtained images were transformed into projections in maximum-intensity mode. All images obtained were analyzed using the ImageJ software (USA).

## Results

### General Morphology of Glochidia

The mature glochidium of *N. douglasiae* is typical of many unionid hooked shells with two dorsally articulated, subtriangular valves, a slightly protruding basal edge forming a straight dorsal line (Figure 2A), and a large single styliform hook at the ventral tip of each valve, covered by lanceolate microspines (Figures 2, 3). A larval thread was present (Figure 2A). Each valve outline was slightly asymmetric, with an anterior margin produced more than the posterior margin. Valves are convex; therefore, the mature glochidial shell appears rounded in certain projections (Figure 2A). At least two sensory hair bundles were visible under each hook (Figure 2B).

For *N. douglasiae* swollen light-yellow outer demibranchs indicated the presence of immature glochidia, while swollen dark-brown outer demibranchs pointed the mature glochidia. Flapping movement of larvae so as the presence of the hooks on ventral edge of each glochidial valve also indicate the mature glochidia. Mature glochidia were registered in late June, early July, and early October. In early May, the early larval stage with the blastula and blastopore was observed (Figures 3A,A1). Immature glochidia with cap-like shells were collected in late May. Some specimens had immature glochidia without hooks in June, although the larval shells had a typical sub-triangular shape. Visible growth lines on the outer surface of the glochidial shells indicated the ongoing larval development, whereas such growth lines were less pronounced or almost invisible on the mature glochidial shells (Figures 3B,B1). The pores in the larval shell were filled with an organic matrix early in the brooding process. Hooks were formed during the final stages of larval development. The thin outer layer of the valve covered the ends of the pores and formed a specific microsculpture. The exterior microsculpture characteristics ranged from loose-looped to tight-looped at different points on the exterior valve surface (Figure 3C). The exterior surface of the valves pitted, especially in the adductor region (central parts of the valves) but was quite smooth along the valve border (Figure 3C). The inner layer was thick and punctate by pores (Figure 3D). The single adductor muscle consisted of fibers stretched from one valve to another (Figure 3E). The large styliform hook on the ventral tip of each valve had a broad base, gradually tapering towards its pointed and slightly inwardly bent end (Figures 3F,F1). Hooks were covered by microstylets or microspines, with a length >1.0 µm, and micropoints (< 1.0 µm), according to Hoggarth (1999). Depending on the locality and collection year, the size of the glochidial shells and hooks varied. Glochidia of *N. douglasiae* from the Russian Far East were 155–185 µm in length and 140–185 µm in height, with ligament 125–140 µm in length and hooks 42–65 µm in length.

### Alpha-Acetylated Tubulin-Lir Larval Structures

To visualize sensory hair cells in *N. douglasiae* glochidia*,* we chose an antibody against acetylated α-tubulin as a marker microtubule included in the multicilia of hair cells (Figure 4). Tubulin antibody stained bright and selective cilia bundles (brush-like) hair and hairless cell bodies and their processes (Figures 4A,A1). All tubulin-stained structures (bodies and processes) were bilaterally symmetrical; the same structures were detected in the left and right valves of the glochidia (Figures 4A2,A3). Tubulin-lir hair cells are arranged specially: three hair cells lie next to each other, and one cell is separated from this bundle of three cells (Figures 4A4,A5). Three hair cells were located under the larval hook and below in solitary hair cells (Figure 4A6). All hair cell processes were collected (in contact) with two non-hair cells on each side of the larva (we called them basal cells, Figure 4A7), which themselves the processes were in contact with each other and ran deep into the body of the larva (Figures 4B,B1). These cells have several processes, and additional staining by antibodies against the neuropeptide FMRFamide (see below) can be identified as afferent neurons collecting information from peripheral tubulin-lir hair sensory cells to larval neurons.

### Muscle System

The muscular system of the glochidia was identified by non-immune staining (phalloidin conjugated with fluorochrome AlexaFluo 555) of F-actin, which was found in large quantities in the smooth muscles of all bivalve mollusks of the fold. Figure 5 shows that the glochidia valves were rigidly connected by a straight hinge and had muscle bundles forming a smooth adductor oriented perpendicular to the valves (Figures 5A–B1). The larval adductor consists of spindle-shaped smooth muscle cells with an elongated concentric nucleus. In addition to the adductor, the glochidia muscular system includes lateral retractors directed to the anterior part (to the hook) and separate posterior and anterior muscle bundles with thin threads running into the ventral part of the larva (Figures 5A,A1). In addition, thin actin filaments in the larval center are visible on the larva in the lateral view (Figures 5B,B1) and are apparently needed to contract the visceral organs.

### The 5-HT-Lir Larval Structures

The serotonin-lir larval system of glochidia consists of two 5-HT-lir neurons located on the posterior part of the larvae (Figures 6A–B4). These 5-HT-lir neurons are connected with each other (Figures 6B2–B4) and possess long neurites running from neurons to the anterior part of the left and right of the valve (Figures 6A–B4). The 5-HT-lir neurites do not physically connect with tubulin-lir sensory hair cells but go through the smooth adductor muscle and are probably involved in muscle relaxation, as is realized in the catch muscle of marine bivalves.

### The FMRFamide-Lir Larval Structure

Unlike the serotonin system of glochidia, the FMRFamide-lir system is more complicated and consists of several neuronal centers comprising neuronal bodies and their neurites in different areas of the larva. The names of the neurons were basal on their spatial location (according to the axes of the larva), for example, anterior, ventral, and dorsal neurons (Figure 7). The FMRFamide-lir system consists of paired anterior, ventral, dorsal, and base neurons (Figures 7A,A1). FMRFamide-lir basal neurons were previously designated as tubulin-lir-basal cells; in this study, we named them neurons because of the neuropeptide FMRFamide expression detected in these cells from the left and right sides of the glochidia valve (Figures 7A2,A3). These neurons are afferent and receive information from sensory (chemosensory) tubulin-lir hair cells responsible for receiving chemical stimuli and/or changing the water density (when animals move in an aquatic environment). FMRFamide-lir paired dorsal neurons connect with double immunopositive (FMRFamide/tubulin)-basal neurons *via* long paired neurites and, in turn, FMRFamide-lir pair-basal neurons connect with ventral neurons *via* neurites (Figures 7A1,B–B3). FMRFamide-lir ventral neurons were located close to tubulin-lir hair cells on the left and right sides of the valves (Figures 7B2,B3).

### The Mutual Arrangement of FMRFamide-Lir, 5-HT-Lir, and Sensory Structures in Glochidia

Triple immunostaining for both sensory and neuronal larval structures showed that antibody labeling of the FMRFa-ir and 5-HT-ir neurons did not indicate colocalization within elements of the nervous system in the glochidia (Figures 8A–B2). The posterior 5-HT-lir is located close to the FMRFamide-lir on the dorsal part of the larva (Figures 8A,B). The 5-HT and FMRFamide–lir neurites from dorsal neurons run together to base neurons (Figures 8A–A2), but transmitters in neurites do not colocalize (Figures 8A–B2). As already reported above, tubulin-lir hair cells express neuropeptide FMRFamide in paired basal cells located on the left and right glochidia valve (Figures 8A,A2).

## Discussion

**FIGURE 9:**
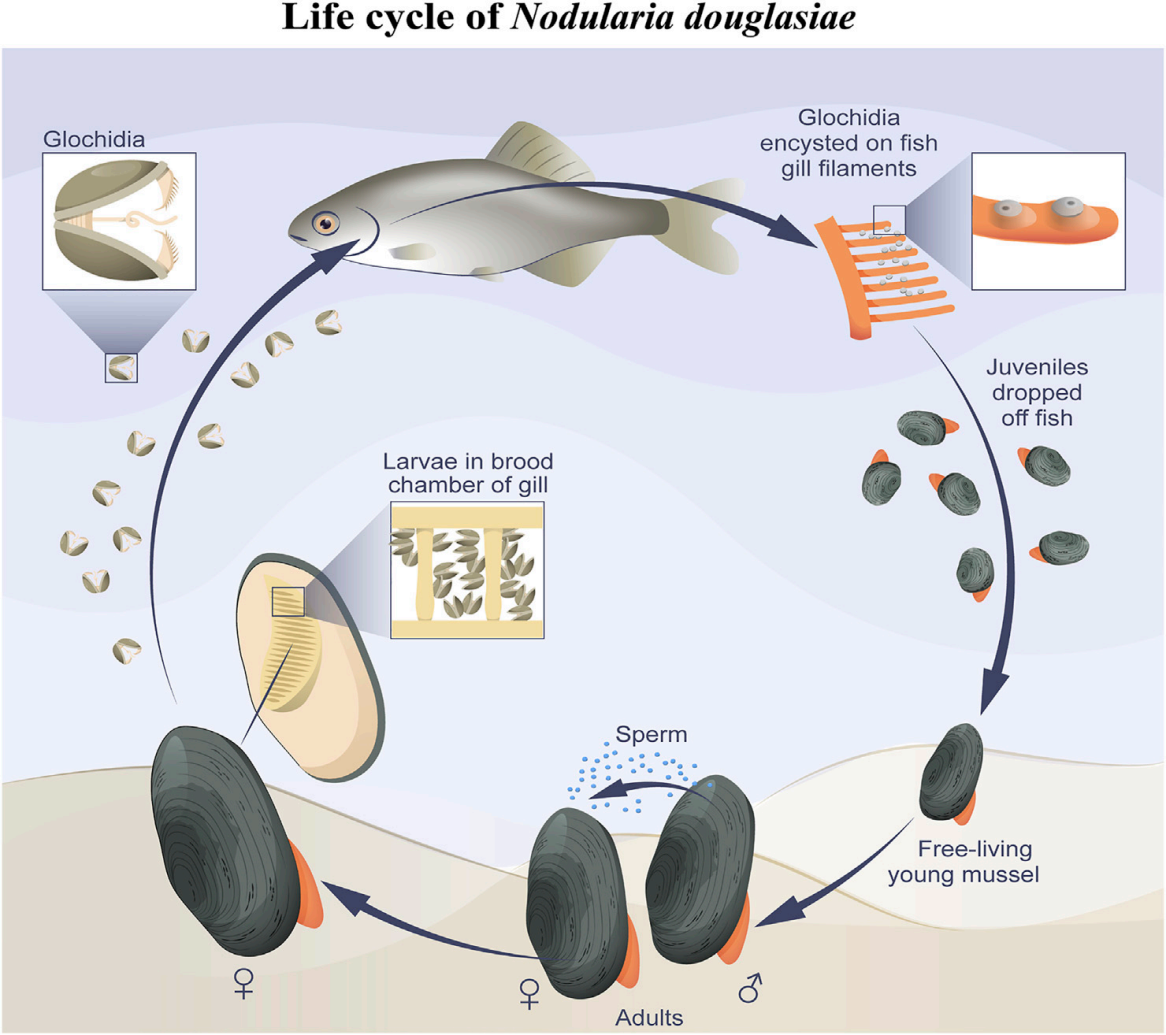
Simplified scheme of *N. douglasiae* life cycle. The main host fish is Amur bitterling *Rhodeus sericeus*.

In this study, we present the first immunocytochemical study on the freshwater mussel *N. douglasiae* and pioneer data on the presence of 5-HT- and FMRFamidergic elements of the nervous system, as well as tubulin for the detection of sensory hair cells in glochidia larvae. Based on morphological data, we can conclude that the nervous and sensory system of *N. douglasiae* glochidia is drastically different from other mollusks and lophotrochozoans by the absence of an apical organ and the location and composition of FMRFamide and 5-HT cells. The Unionoidea species life cycle lacks larval stages such as trochophores, veligers, and pediveligers inherent to marine lamellibranch species (pecten, oysters, sea mussels, and clams). Instead of planktotrophic larvae, some Unionida have a larval stage known as glochidium, whereas others have a lasidium (Hoggarth, 1999; Wächtler et al., 2001; Sayenko, 2006). In contrast to the larvae floating freely and actively in the water column, glochidia attach to and encyst in fish and move, thus providing their dispersal (Nezlin et al., 1994; Castrillo et al., 2020). The general scheme of the *N. douglasiae* life cycle is shown in Figure 9 and is characteristic of all Unionidae.

The appearance of glochidia is an important episode in bivalve evolution, which is the colonization of freshwater environments by an ancestral unionoid species during the Triassic period, obtaining access to a bivalve-free ecosystem. New habitats and specific environmental conditions could have been triggered by the evolution of a novel development mode using glochidium-type larvae, with fish as intermediate hosts (Graf, 2000; Giribet, 2008).

For a long time, glochidia were studied using only a light microscope (Surber, 1912; Inaba, 1941, 1964; Wood, 1974; Antonova and Starobogatov, 1988), and because of their very small size, only dimensional characters were accessible. Using scanning electron microscopy, new minute features of glochidia have been obtained (Giusti, 1973; Kinzelbach and Nagel, 1986) and used to interpret the relationships among taxa (Kondo, 1982, 1998; Hoggarth, 1999; Sayenko, 2006; Sayenko et al., 2021). The morphological features of mature glochidia have been used to classify Unionidae (Kondo, 1982, 1998; Hoggarth, 1999; Sayenko et al., 2021). DNA data analysis, together with differences in glochidia morphology, allowed the separation of three closely related Asian genera with uncertain taxonomic status, namely *Nodularia*, *Middendorffinaia*, and *Inversiunio* (Lopes-Lima et al., 2020; Sayenko et al., 2021).

There are few studies on the internal morphology of glochidia of freshwater mollusks (Jupiter and Byrne, 1997; Araujo and Ramos, 1998; Fisher et al., 2002). The necessarity of using special complicated methods to prepare glochidia specimens in order to preserve details of their soft anatomy for photography under scanning electron microscopy makes such investigations less accessible compared to the study of glochidia shells morphology. The anatomy and morphology of the glochidium of *Margaritifera auricularia* (Unionoidea: Margaritiferidae) have been studied using optical and electronic microscopy and histological techniques (Araujo and Ramos, 1998). The following cells and tissues of glochidia were described: muscle, oral plate in the center of the larva, and ventral plate (anlage of the foot), covered with lateral pits with dense cilia. Two pairs of hair sensory bundles were observed at the edges of the valves. No larval organs or even their rudiments, such as the pericardium, kidneys, heart, or nerve structures, develop, as concluded by Araujo and Ramos (1998).

Little is known about glochidia metamorphosis into juveniles, as investigations have mainly focused on the morphology of glochidial shells. Metamorphosis encompasses a few distinct stages, with some variation between the investigated taxa. The first involves the degeneration of the single larval adductor muscle and the formation of the characteristic mushroom body, when the inner larval mantle cells gradually hypertrophied and protruded into the mantle cavity so these cells were grouped together and appeared as a mushroom body when viewed from the frontal section. The final stage involves the formation of major anatomical structures and organ systems in the juveniles (Wood, 1974; Fukuhara et al., 1990; Fisher et al., 2002; Chumnanpuen et al., 2011).

Morphological development of glochidia of freshwater pearl mussel, *Hyriopsis* (*Hyriopsis*) *bialatus* in artificial media showed that the ventral plate of glochidium is an anlage of the foot, lateral pits are an anlage of the gills, and the oral plate or endothermic sac is the beginning of the digestive tract. The digestive tract begins with the formation of the oral cavity by invagination of the oral plate and a tube under the growing foot. Several controversial aspects of organogenesis have been inferred, for example, the formation of *de novo* anterior and posterior juvenile adductors (Chumnanpuen et al., 2011).

### Hair Cells

Hair cells are mechanoreceptor cells with a bundle of cilia involved in the cells’ ability to respond to mechanical stress to the membrane. In invertebrates, mechanoreceptors are comparable to the apical organ (AO), whose cells also often have a multiciliary nature and presumably act as chemosensory and/or mechanosensory organs in the development of Cnidaria, Lophotrochozoa, Echinodermata, and Hemichordata (Sinigaglia et al., 2015). Hair cells may act as primary sensory cells (specialized neurons with their axons, capable of perceiving stimuli and generating a nerve impulse) and secondary sensory cells that form synapses with sensory neurons transmitting impulses. In the lophotrochozoan line, unique hair cells with many kinocilia but without stereovilli have been described. Among invertebrates, marine mollusk larvae trochophores and veligers have multiciliary AO, and freshwater mollusk larvae glochidia have sensory hair cells that develop from specialized ectodermal cells and contain several cilia on the larval mantle.

The bivalve *Anodonta arcaeformis* (Unionidae) has two types of hair cells in its larval mantle (Jeong et al., 1992). Interestingly, the first type of cells has a bundle of protruding hairs in three isolated and highly specialized cells in the mantle, presumably perceiving chemical stimuli (Wood, 1974). The function of another type of hair cells located on the posterior edge near the lateral pits remains unknown. The number of the second type of hair cells varies and is sometimes used for taxonomic classification. For example, two pairs of sensory cells have been identified in *Margaritifera margaritifera*, and *M. auricularia*, rather than the four found in most other Unionoidea glochidia (Pekkarinen and Englund, 1995; Pekkarinen and Valovirta, 1996). However, the number of these cells is not always strictly a taxonomic indicator. For example, in the genus *Hyriopsis*, the number and distribution of this type of hair cells in the closely related species *H. bialatus* and *H. myersiana* are different (Areekijseree et al., 2002), and therefore, this is not a reliable criterion.

**FIGURE 10:**
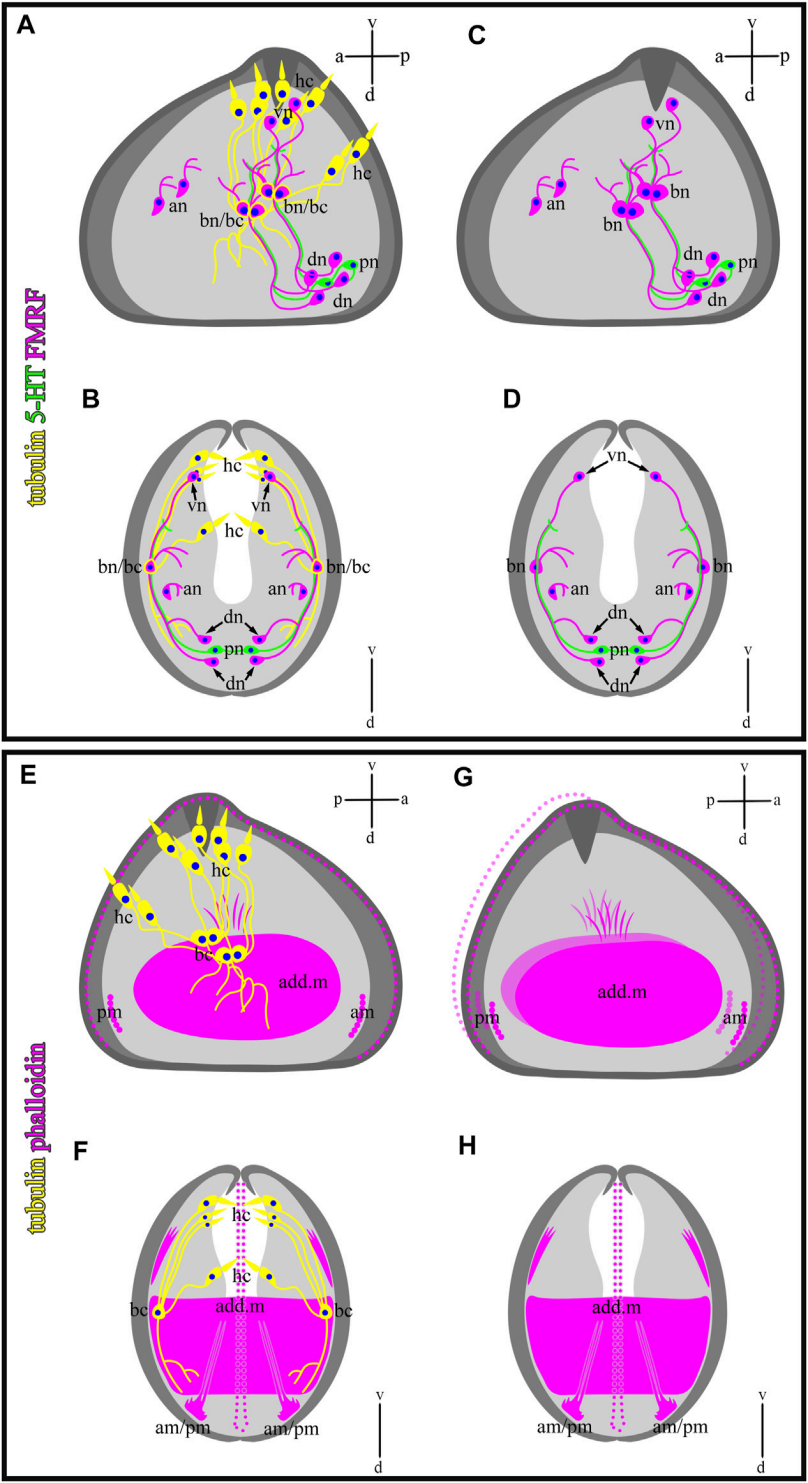
Schematic drawings of a mutual distribution of sensory (tubulin-lir) and nervous (serotonin- and FMRFergic) system **(A,B)**, serotonin-lir and sensory system **(C,D)**, muscle (phalloidin) and sensory system **(E,F)**, and muscle system alone **(G,H)** of glochidium of *Nodularia douglasiae* (profile and full-face). **(A,C,E,G)** Vertical v-d labelled line refers to ventro-dorsal body axes and horizontal line a-p labelled line refers to anterior-posterior body axes of larva. **(B,D,F,H)** Vertical v-d labelled line refers to ventro-dorsal body axes. Abbreviations: add. m, adductor muscle; an, anterior neurons; am, anterior muscle; bn, basal neurons; dn, dorsal neurons; hc, hair cells; lr, larval retractors; pc, posterior cells, pm, posterior muscle; vn, ventral neurons.

In this study, we showed that glochidia of the freshwater bivalve *N. douglasiae* have four paired hair cells, of which three cells and their processes are located tightly at each valve, and one pair is kept apart more dorsally (Figure 10). We first identified hair cells and described their morphology using the whole mounting method of immunostaining glochidia. We found hair cell connections with non-ciliated tubulin/FMRFamide-lir cells, which communicate with other neurons *via* neurites (Figure 9). These hair mantle cells are chemosensory cells that are extremely important for host attachment (Wood, 1974). It is unknown whether sensory hair cells express neurotransmitters other than 5-HT and FMRFamide. In vertebrates, the cholinergic chemosensory cell in the trachea, which expresses the acetylcholine-synthesizing enzyme choline acetyltransferase, is linked to afferent nerve fibers expressing nicotinic acetylcholine receptors (nAChR), resulting in organ-specific reflex responses (ChAT) (Krasteva et al., 2011). Сatecholamine-containing cells in mollusks (gastropods and bivalves) are found in cephalic sensory organs (tentacles, rhinophores, lips), in the foot and mantle as well as in mollusk larvae, and it has been suggested that they may mediate mechanoreception and a generalized chemosensory function (Croll et al., 1997; Wyeth and Croll, 2011; Voronezhskaya and Croll, 2015). In addition, FMRFamide-lir is synthesized in the neuron and can innervate adductors and regulate their contraction; low concentrations of FMRFamide relax ACh-induced catch-tension, whereas high concentrations cause contraction (Muneoka and Saitoh, 1986). Apparently, the contraction of the larval smooth adductor muscle and its reactive movement is realized initially by sensory ciliated hair cells that come into contact with neurons, the processes of which innervate smooth muscles and cause their contractions.

### Nervous System

Most morphological studies on the structure and development of glochidia claim that the nervous ganglia are not present in glochidia. For example, in the glochidia of *Hyriopsis bialatus* and *Anodonta cygnea* (Unionidae), as well as *Margaritifera auricularia* (Margaritiferidae), it is claimed that the nervous system of these species is absent (Wood, 1974). Although several studies have not revealed neuronal structures in glochidia of different species, there is evidence that *Anodonta* glochidia have developed a cerebral ganglion and sometimes a visceral ganglion (Harms, 1909). Suppose we really accept the absence of nerve elements in glochidia. In that case, the following question arises: how is the regulation of the contraction of the main (adductor) and minor muscles (muscle cells on the periphery) of glochidia, which must actively move in the water column with the help of jet movement created by the contraction of catch muscles, as is realized in marine larvae-veligers of bivalve mollusks (Oditsova and Dyachuk, 2009; Dyachuk et al., 2012). It is assumed that the functions of the nervous system can be partly seized by hair cells in the glochidial mantle (Pekkarinen and Valovirta, 1996), providing the tactile response of the larva and probably acting as chemosensory neurons that regulate the contraction of smooth muscles.

We have shown that the 5-HT and FMRFamide-lir nervous systems are present in the freshwater bivalve *N. douglasiae*. The larva’s nervous system is represented by two 5-HT-ir cells and processes, and four paired FMRFamide-lir neurons connected by neurites (Figure 10), forming a complex system regulating the larva’s behavioral patterns. A thorough search of published data on the nervous system of glochidia led us to the study by Pekkarinen and Valovirta (1996), who showed in glochidia of two anodontins, *Anodonta anatina* and *Pseudanodonta complanata,* three pairs (cerebral, pedal, and visceral) of rudimentary ganglia using histochemical staining and visualized by light and scanning electron microscopy (Pekkarinen and Valovirta, 1996). The author claims that the cerebral ganglia (CG) are visible in the lateral parts of the oral plate, partially overlapping with the rudimentary digestive diverticula. The primordia of the visceral ganglia (VG) are closely associated with the posterior side of the lateral pits of glochidia, and another pair of cell groups is associated more medially with the posterior wall of the lateral pits, which may be identified as the primordia of the pedal ganglia (PG). Based on the morphology of the non-stained nervous tissue (ganglia were not stained with methylene blue, azo dye, or cobalt sulfide during alkaline phosphatase staining) (Pekkarinen and Valovirta, 1996), it is difficult to distinguish nerve ganglia from neighboring camps and their belonging to one or another ganglion in glochidia.

The development and even the presence of nerve nodes have been the subject of discussions of malacologists for 120 years. Early studies described the formation of CG from ectodermal thickenings and invaginations to the growth plate in *Anodonta* glochidia, and the pedal ganglia developed from the ectodermal layer and did not develop earlier than the parasitic phase (Lillie, 1895; Harms, 1907, 1909). Visceral ganglia probably develop from the pockets of the lateral pits (Schierholz, 1889; Pekkarinen and Valovirta, 1996). In contrast, on the same species Kinzelbach and Nagel (1986) reported that no ganglia were visible in glochidia.

Owing to the lack of specific immunostaining morphological data on the structure of the nervous system of glochidia of other freshwater mollusks, it is impossible to conduct a comparative analysis of the similarities and differences in the structure of the nervous systems of Unionidae.

## Data Availability

The raw data supporting the conclusion of this article will be made available by the authors, without undue reservation.
